# Ultrasound-guided high-intensity focused ultrasound for symptomatic uterine fibroids: clinical outcome of two European centers

**DOI:** 10.1007/s00330-024-11230-4

**Published:** 2024-11-29

**Authors:** Sara Dohmen, Florian Recker, Yoana Ivanova, Holger M. Strunk, Tolga Tonguc, Olga Ramig, Marcus Thudium, Judith M. Stader, Rupert Conrad, Markus Essler, Eva-Katharina Egger, Alexander Mustea, Grigor A. Gortchev, Dobromir Dimitrov, Milka Marinova

**Affiliations:** 1 Department of Nuclear Medicine, University Hospital Bonn, Bonn, Germany; 2 Department of Rаdiology, University Hospital Bonn, Bonn, Germany; 3 Department of Gynaecology and Gynaecological Oncology, University Hospital Bonn, Bonn, Germany; 4 Department of Obstetrics and Prenatal Medicine, University Hospital Bonn, Bonn, Germany; 5 St. Marina University Hospital, Medical University Pleven, Pleven, Bulgaria; 6 University of Bonn, Bonn, Germany; 7 Department of Anesthesiology, University Hospital Bonn, Bonn, Germany; 8 Department of Psychosomatic Medicine and Psychotherapy, University Hospital Muenster, Muenster, Germany; 9 Department of Surgical Propedeutics/HIFU Center University Hospital St. Marina, Medical University Peleven, Pleven, Bulgaria

**Keywords:** High-intensity focused ultrasound (HIFU), Symptomatic uterine fibroids, Fibroid-related symptoms, Health-related quality of life, Non-invasive treatment option

## Abstract

**Objectives:**

The aim of this study is to assess the clinical outcome and mid-term efficacy of ultrasound-guided high-intensity focused ultrasound (USgHIFU) as a treatment for symptomatic uterine fibroids at two major European HIFU centers.

**Materials and methods:**

This bi-center longitudinal clinical study involved the treatment of 100 patients with symptomatic uterine fibroids using USgHIFU (*n* = 59 in Germany, *n* = 41 in Bulgaria). Clinical outcomes were evaluated at 6 weeks, 6 months, and 1 year follow-up utilizing the uterine fibroid symptoms-quality of life questionnaire for fibroid-related symptoms and health-related quality of life as well as MRI imaging for determining the fibroid volume.

**Results:**

The mean fibroid volume reduction rate was 33.2 ± 22.9%, 51.3 ± 24.2%, and 59.1 ± 28.0% at 6 weeks, 6 months, and 1 year, respectively (each *p* < 0.001). The mean symptom severity score decreased from 43.9 ± 18.8 at baseline to 35.4 ± 18.2 at 6 weeks, 31.1 ± 20.0 at 6 months, and 23.1 ± 14.0 at 1 year (each *p* < 0.001). The mean QOL score improved from 56.5 ± 23.4 at baseline to 65.4 ± 22.2 at 6 weeks, 72.5 ± 19.5 at 6 months, and 79.4 ± 15.3 at 1 year (each *p* < 0.001). No major complications were observed, though two patients experienced temporary sciatic nerve irritation following the procedure. Four patients had pregnancies and deliveries without any complications after USgHIFU therapy.

**Conclusion:**

To our knowledge, this is the first longitudinal study conducted in two major European HIFU centers that reveals the clinical efficacy of USgHIFU ablation on symptomatic uterine fibroids. Our results confirm that USgHIFU is a non-invasive approach with a low risk of complications, offering an innovative treatment option for affected women.

**Key Points:**

***Question*** *To evaluate mid-term clinical efficacy and safety of US-guided high-intensity focused ultrasound (HIFU) for treating symptomatic uterine fibroids and patient outcomes across two European centers.*

***Findings*** *US-guided HIFU treatment resulted in significant fibroid volume reduction (up to 59.1% after 1 year) improving symptoms and quality of life with no major complications.*

***Clinical relevance*** *This prospective longitudinal study provides preliminary data assessing mid-term efficacy and clinical outcomes of ultrasound-guided HIFU. It is shown to be a low-risk, non-invasive treatment option for symptomatic uterine fibroids that reduces fibroid size and improves patients’ quality of life.*

## Introduction

Uterine fibroids are the most common benign tumors in women of reproductive age. Although benign, they are associated with significant morbidity in 20–50% of women. Symptoms may include menorrhagia, metrorrhagia, pelvic pain and pressure, urinary urgency, and even infertility [1]. Recent consensus conferences recommend that the treatment of uterine fibroids should be approached interdisciplinary, with thorough consultation on all possible treatment options [2]. Therapeutic options for symptomatic uterine fibroids are classified into non-uterus-preserving and organ-preserving procedures. Non-uterus-preserving options include various forms of hysterectomy, while organ-preserving options include open, laparoscopic, and transcervical myomectomy, pharmacological therapies, radiofrequency ablation, and uterine arterial embolization (UAE) [3, 4]. In recent years, high-intensity focused ultrasound (HIFU) has shown promise as an effective and low-risk treatment option for symptomatic uterine fibroids [3–6]. Currently, HIFU treatment is guided either by ultrasound (USgHIFU) or magnetic resonance imaging (MRgHIFU) [7]. USgHIFU devices use diagnostic ultrasound to locate the target region and monitor the treatment response in real time. Successful tissue ablation is indicated by grayscale changes and cavitation observed on real-time B-mode ultrasound images after each exposure. MRI-guided systems apply MRI to determine target regions for therapeutic ultrasound, and indirect MRI thermometry confirms target position and measures therapeutic response.

The primary basis of HIFU therapy lies in the induction of thermally induced coagulation necrosis in the target tissue. This is accomplished through focusing ultrasound waves in the fibroid, resulting in localized heating over 60 °C and consequent tissue destruction [7, 8]. Further mechanisms of action entail direct mechanical effects, specifically the generation of shear forces generated during the compression and expansion of the target tissue which leads to damage of cell membrane [9]. The fundamental advantage of HIFU therapy, in comparison to other local ablative treatments, is its non-invasiveness and low complication rate. The treatment does not require the use of needles, probes, or electrodes [10], rendering HIFU an alluring treatment choice, especially for women who want to become pregnant in future. The objective of this bi-center, prospective, observational study was to evaluate the mid-term efficacy of USgHIFU for managing symptomatic uterine fibroids. To our knowledge, this is the first study to compare clinical outcomes of 100 patients treated with USgHIFU at two major European HIFU centers. Treatment success was evaluated using objective measures including changes in fibroid volume, health-related quality of life (HRQoL) and symptom severity during 1 year follow-up period compared to baseline. In addition, contrast-enhanced T1W imaging was used to determine the non-perfused volume (NPV, %) of the fibroids in study group 1 (*n* = 65) and the fibroids to be treated were categorized according to the Funaki classification [11].

## Patients and methods

**Fig. 1:**
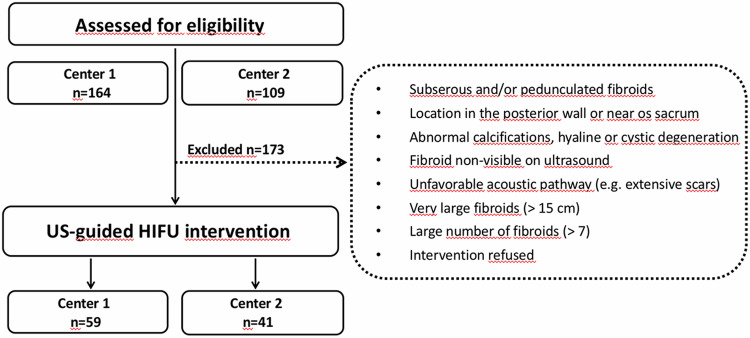
Study consort diagram: assessment of eligibility

A total of 100 patients with symptomatic uterine fibroids received USgHIFU treatment from 2017 to 2019 in a prospective, observational clinical study conducted at two cites. Specifically, 59 patients were treated at Germany’s University Hospital Bonn, and 41 patients received treatment at St. Marina Hospital in Bulgaria. Eligibility and clinical indication were assessed by an interdisciplinary committee for each patient (Table 1, Fig. 1). Patients with symptomatic transmural uterine fibroids were included in this study (FIGO types 2–5 fibroids according to the classification of the International Federation of Gynecology and Obstetrics [12]).

**Table 1:** Selection criteria for US-guided HIFU treatment of symptomatic uterine fibroids

Inclusion criteria	Exclusion criteria
Age ≥ 18 years	Pedunculated subserosal fibroids
Written informed consent	Fibroids with severe calcifications or hyaline degeneration
Distance between skin surface and deepest tumor regions of max. 11 cm in prone position	Suspected uterine malignancy clinically or by imaging
Safe acoustic access path to the lesion	Non-eligibility for conscious sedation
Fibroid-related symptoms	Lesion not sufficiently visible on ultrasound, e.g., due to extensive scarring in the acoustic pathway
Fibroid size ≤ 12 cm	Pregnancy
Eligibility for prone positioning and sedation	Acute inflammation

The study received approval from the local ethics committees at the two sites (Medical Faculty, University of Bonn, Germany, no. 295/19; Medical University of Pleven, Bulgaria, no. 639-kenid) and was performed in accordance with the Declaration of Helsinki. Written informed consent was obtained from all participants. In the following, patients treated at the German HIFU center are designated as group 1 (G1), while those treated at the Bulgarian HIFU center are designated as group 2 (G2). Unless otherwise indicated, data refer to the entire patient cohort (*n* = 100) of the two HIFU centers. Table 2 presents an overview of the demographic and baseline clinical characteristics of HIFU-treated patients in both centers.

**Table 2:** Baseline demographic and clinical characteristics of patients treated with US-guided HIFU

	Total	Patient group 1^a^	Patient group 2^b^
Patients, *n*	100	59	41
Age	42.1 ± 6.2	43.0 ± 6.2	40.6 ± 5.9
	(25.0–57.8)^c^	(28.9–57.8)	(25.0–51.0)
Treated fibroids, *n*	119	78	41
Treated fibroids per patient, *n*			
1	87 (87%)	46 (78.0%)	41 (100%)
2	7 (7.0%)	7 (11.9%)	
≥ 3	6 (6.0%)	6 (10.2%)	
Severity of fibroid-associated symptoms^d^			
Mild	31.9%	20.8%	46.3%
Moderate	53.2%	58.5%	46.3%
Severe	14.9%	20.8%	7.3%

^a^ Patient group 1, treated by USgHIFU at Center 1

^b^ Patient group 2, treated by USgHIFU at Center 2

^c^ Mean ± standard deviation (range)

^d^ Severity of fibroid-associated symptoms based on Symptom Severity Score (SSS): mild (0–33.3), moderate (33.4–66.6), severe (66.7–100)

### HIFU ablation

For US-guided ablation, the Focused Ultrasound Tumor Therapeutic System (JC Chongqing HAIFU Technology), equipped with a 1–8 MHz ultrasound imaging device (MyLab 70, Esaote), is utilized. The same machine is used at both sites for fibroid ablation. Therapeutic ultrasound is generated by a ceramic transducer (diameter 20 cm, focal length 15 cm, frequency 0.8 MHz). The therapeutic parameters (sonication time, total energy, average power and energy/volume) are listed in Table 3. Pre-interventional screening includes an in-depth review of the patient’s medical history, physical and gynecological examination, symptom assessment, and laboratory tests. Contrast-enhanced MRI and transabdominal sonography are conducted on the pelvic region up to 8 weeks before undergoing USgHIFU treatment. Bowel preparation, including a 12-h fast and laxative use, is implemented the day before to reduce bowel gas and minimize any potential thermal side effects. Prior to initiating USgHIFU treatment, specific factors such as fibroid size and proximity to adjacent structures at risk are identified and carefully considered during patient enrollment to minimize the risk of severe adverse events. Additionally, the skin in the acoustic pathway is shaved and degreased. The skin of the anterior abdominal wall is degassed with a special device immediately before the treatment to remove the smallest residual air bubbles from the skin area and prevent skin burns. To optimize the acoustic window, a degassed water-filled balloon is inserted between the patient’s anterior lower abdominal wall and the transducer during the procedure. The USgHIFU treatment is conducted with the patient under conscious intravenous sedation. During ablation procedure, a visual NPV greater than 70% of the predominant fibroid was attempted.

**Table 3:** Therapeutic parameters of US-guided HIFU in patients with uterine fibroids

	Patient group 1^a^	Patient group 2^b^	Total
Sonication time (s)^c^	1023 ± 454	407 ± 173	799 ± 479
	(277–2013)^d^	(119–719)	(119–2013)
Sonication time (min)	17.05 ± 7.57	6.78 ± 2.88	13.32 ± 7.98
	(4.62–33.55)	(1.98–11.98)	(1.98–33.55)
Total energy (kJ)	283.9 ± 153.3	111.8 ± 59.6	221.5 ± 151.9
	(44.4–586.9)	(28.8–232.9)	(28.8–586.9)
Energy/volume (kJ/mL)	7.43 ± 8.47	4.84 ± 18.02	6.45 ± 12.88
	(0.61–46.95)	(0.06–102.80)	(0.06–102.80)

^a^ Patient group 1, treated by USgHIFU at Center 1

^b^ Patient group 2, treated by USgHIFU at Center 2

^c^ Time from first to last sonication

^d^ Mean ± standard deviation (range)

For planning and HIFU ablation, a sagittal scanning mode is used; US energy is delivered to a circumscribed focal area using a dot mode (an oval-shaped focus of 1–3 mm in width and 8–15 mm in length). Repeated cycles of 1 s sonication followed by a 3 s break are delivered at each focal point. In case of visible grayscale changes in the target area suggesting effective ablation or after a minimum of 50 s sonication time the transducer is moved to the next focal zone in the same slide, then in adjacent slides in order to achieve volume ablation. Thus, multiple adjacent lesions on bordering layers produce linear and discoidal necrosis patches, allowing the entire fibroid area to be ablated as a volume unit.

During each treatment session, a contrast-enhanced ultrasound (CEUS) was performed at the beginning and toward the end of the intervention to assess the blood flow to the fibroids. If the CEUS showed an insufficient non-perfused area towards the end of the ablation, visually affecting less than 50% of the fibroid volume, the ablation was continued in the same session. The therapeutic parameters, including sonication time, total energy, and energy per mL fibroid volume, are shown in Table 3.

USgHIFU was performed at both sites by a multidisciplinary team comprised of an anesthesiologist, nurses, and certified physicians (radiologists, gynecologists, surgeons), who had experience in treating a minimum of 20 patients during specialized tumor therapeutic training with the USgHIFU system. Therefore, as a result of their training and experience, both teams implemented the same clinical protocol for the selection of patients and the administration of treatment. During HIFU, patients lie in a prone position and provide ongoing feedback to prevent damage to the leg nerves. Side effects such as abdominal pain or vaginal bleeding are monitored, categorized according to the Clavien-Dindo classification, and followed until they subside. Most patients were discharged home within 6 h; a few stayed overnight, depending on their condition.

### Follow-up

For MRI examinations of the pelvis, different machines (Philipps, Siemens, and GE) were used. In most cases (ca. 90%), MRI was performed at 1.5 Tesla at both sites; the rest was conducted at 3-Tesla scanners. Different coils were used for imaging with a preference for pelvic phased array coils and multi-channel body coils. Patient positioning was uniform (head first). Following, series of the female pelvis with angulations to the uterus were conducted at a minimum: axial and sagittal T2W (weighted) images; sagittal and axial, native T1W; diffusion-weighed imaging; sagittal and axial, late contrast-enhanced (CE) T1W series. The ratio of fibroid volumes at each post-intervention time point (6 weeks, 6 months, and 1 year) to the corresponding baseline values was used to calculate the reduction in fibroid volume and volume reduction rate. Exact fibroid diameters in three planes (anterior-posterior, cranio-caudal, right-left) were measured on MR images using T2W and contrast-enhanced T1W sequences. In order to estimate fibroid volumes, volume formula of an elongated ellipsoid (V = a*b*c*(π/6)) was used. The objective primary lesion response was assessed by determination of non-perfused volume ratio (NPV, %) on contrast-enhanced T1W imaging and was defined as the ratio of NPV in first post-HIFU MRI compared to the whole fibroid volume. Patients’ health-related quality of life (HRQoL) and symptom severity score (SSS) were evaluated at 6 weeks, 6 months, and 1-year follow-up utilizing the uterine fibroid symptoms-quality of life questionnaire compared with baseline values recorded before the HIFU intervention [13, 14]. In addition, in G1, the objective response of the primary lesion was evaluated by determining the non-perfused volume ratio (NPV, %) on contrast-enhanced T1W imaging. This ratio was defined as the proportion of the non-perfused volume on the first MRI scan after HIFU treatment (within the first week post-intervention) to the total fibroid volume of all treated fibroids (up to three fibroids per patient in G1). G1 fibroids were also graded using the Funaki classification, which assesses fibroid intensity on MRI images relative to skeletal muscle and myometrium. Funaki type 1 fibroids appear hypointense compared to skeletal muscle, type 2 fibroids are hypointense to the myometrium and hyperintense to skeletal muscle, while type 3 fibroids are hyperintense to the myometrium [11, 15]. Complications or adverse events following USgHIFU treatment were categorized using the Clavien-Dindo classification. This classification system provides a standardized approach to categorizing and grading surgical complications or adverse events according to their severity [16].

### Statistical analysis

Statistical analysis was conducted using Stata (version 16.1, StataCorp: StataCorp LP). To assess the comparability of baseline volumes between the two study groups, a parametric *t*-test was employed after an upstream homoscedasticity test (Bartlett’s) and a non-parametric Mann-Whitney U-test (Wilcoxon rank-sum test). Follow-up values (fibroid volume, HRQoL, and SSS) were compared to their respective baseline values utilizing mixed linear data models allowing for missing data. Bivariate associations among intervention variables were investigated through Spearman’s correlations.

## Results

According to the inclusion and exclusion criteria (Table 1), a total of 100 out of 273 (36.6%) patients with symptomatic transmural uterine fibroids (FIGO types 2–5 fibroids according to the FIGO classification [12]) were included in this study and received treatment utilizing USgHIFU. In this cohort of 100 patients, divided into G1 (*n* = 59) and G2 (*n* = 41), aged between 25.0 and 57.8 years (with a mean age of 42.1 ± 6.2 years), successful USgHIFU treatment was performed for up to three fibroids per patient. In G2 only the predominant fibroid was treated and evaluated during the follow-up period. The most prevalent initial symptoms were hyper-/dysmenorrhea in 94.8% of cases, anemia-related fatigue in 93.1%, urinary urgency and frequency in 84.5%, pelvic pain or pressure in 82.8%, and infertility in 54.2%. Prior to undergoing HIFU therapy, symptoms associated with fibroids were categorized as mild (SSS 0–33.3) in 31.9% of cases, moderate (SSS 33.4–66.6) in 53.2% of cases, and severe (SSS 66.7–100) in 14.9% of cases.

### Fibroid volume

**Fig. 2:**
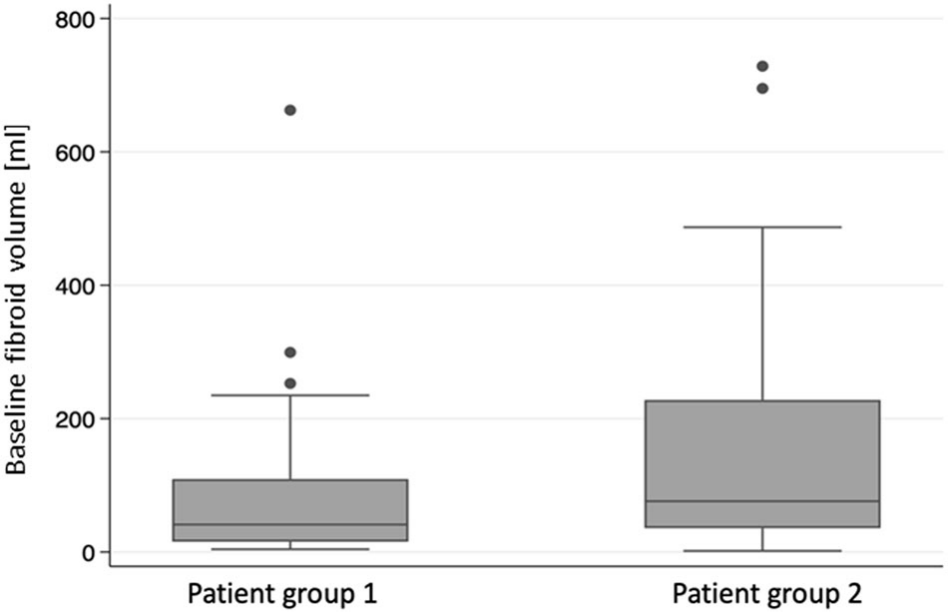
Initial pre-interventional fibroid volume (mL) in group 1 (G1) and group 2 (G2). Measurements were made on T2-weighted MRI

**Fig. 3:**
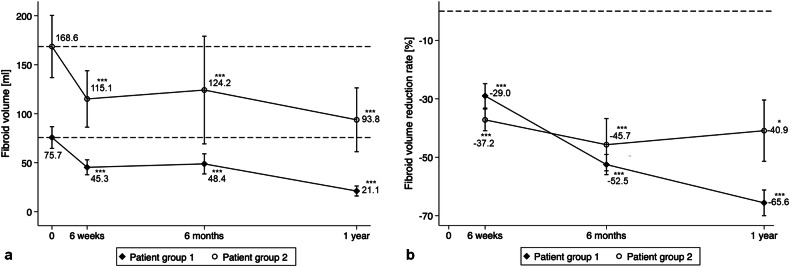
Uterine fibroid volume (mL) (**a**) and fibroid volume reduction rate (%) (**b**) after USgHIFU intervention compared to baseline in G1 and G2 are shown; *p*-values refer to comparison with baseline (*p ≤ 0.05; **p < 0.01; ***p < 0.001)

**Fig. 4:**
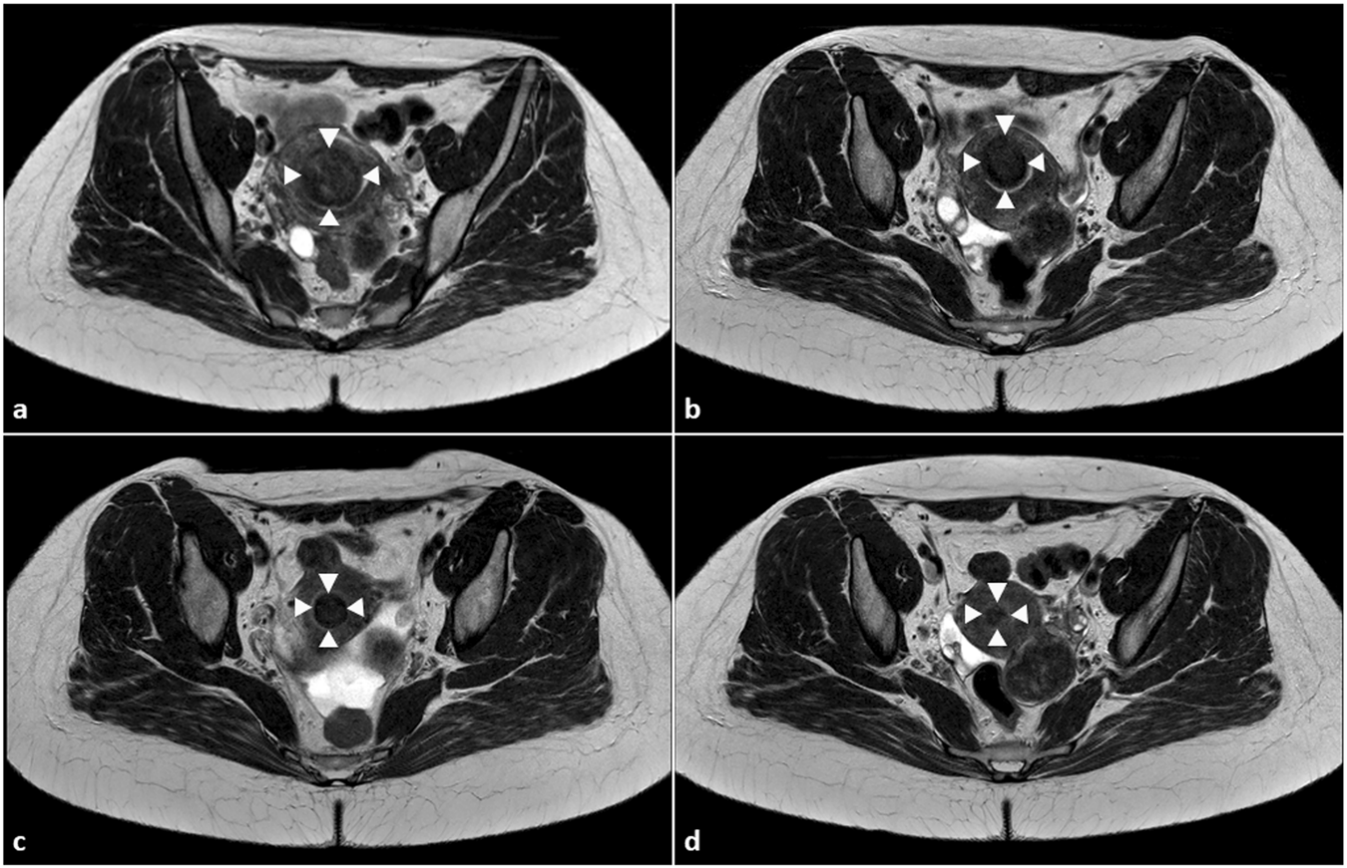
MRI images (transverse T2-weighted sequences) of the pelvis of a 41-year-old premenopausal patient are shown. The patient presented with severe fibroid-associated symptoms (hypermenorrhea, dysmenorrhea, anemia, pelvic pain and feeling of pressure). The predominant target fibroid was located in the fundus of the uterus. In the 1-month follow-up, a significant reduction in symptoms and fibroid volume was observed. **a** Large predominant target fibroid in the fundus of the uterus (white arrows) prior to HIFU treatment. **b** Significant fibroid shrinkage was observed at 3 months with a 54% reduction in lesion volume. **c**, **d** Fibroid volume reduction of approximately 85% at 6 months (**c**) and 94% at 1 year (**d**) post-HIFU

**Fig. 5:**
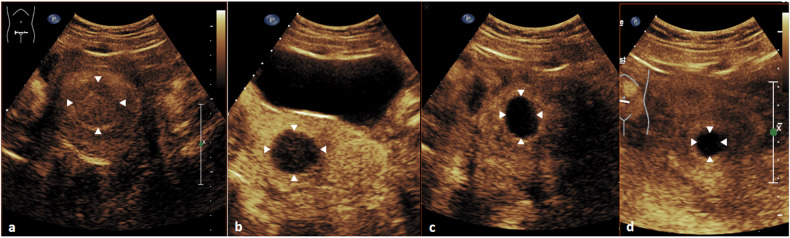
Contrast-enhanced ultrasound images of the uterus of a 41-year-old premenopausal woman with symptomatic uterine fibroids are shown. The predominant target fibroid in the fundus of the uterus was treated with USgHIFU. **a** Predominant target fibroid in the fundus of the uterus (white arrows) with contrast enhancement prior to HIFU treatment. **b** Immediately after HIFU ablation (2 h post-HIFU), a large area without contrast enhancement is shown (white arrows). **c**, **d** A significant reduction in fibroid volume of approximately 54% after 3 months (**c**) and 85% after 6 months (**d**) compared to the initial lesion volume was observed

For follow-up, MRI was used to assess volume changes in the predominant (G2) or three predominant (G1) fibroids. The average initial volume of the fibroids was 105.5 ± 140.2 mL (range 1.6–728.4 mL). The initial volumes were dissimilar between G1 (75.7 ± 96.5 mL) and G2 (168.6 ± 190.7 mL) (*t*-test, *p* = 0.008; U-test, *p* = 0.003; Fig. 2). Follow-up assessments demonstrated a mean reduction rate in volume of 33.2 ± 22.9%, 51.3 ± 24.2%, and 59.1 ± 28.0% at 6 weeks, 6 months, and 1 year following the intervention, respectively (each *p* < 0.001). One year post-HIFU, there was a mean reduction in fibroid volume from 75.7 ± 96.5 mL to 21.1 ± 27.0 mL (65.6 ± 23.3%, *p* < 0.001) in G1 and from 168.6 ± 190.7 mL to 93.8 ± 103.0 mL (40.9 ± 33.1%, *p* < 0.001) in G2 (Table 4, Fig. 3). Representative images of a patient with symptomatic uterine fibroids, who underwent USgHIFU treatment are shown in Figs. 4 and 5.

**Table 4:** Fibroid volume (mL) and corresponding volume reduction rate (%) at baseline and 6 weeks, 6 months and 1 year after the US-guided HIFU intervention (measurements on T2-weighted MRI; number of observations at the corresponding time points: 112, 66, 56, 46)

	Total		Patient group 1^b^		Patient group 2^c^	
	Volume (mL)	Reduction rate (%)	Volume (mL)	Reduction rate (%)	Volume (mL)	Reduction rate (%)
Baseline	105.5 ± 140.2^a^ (1.6–728.4)		75.7 ± 96.5 (4.0–662.5)		168.6 ± 190.7 (1.6–728.4)	
6 weeks	81.2 ± 128.2 (0.9–710.5)	33.2 ± 22.9 (28.0–92.9)	45.3 ± 43.2 (1.7–188.1)	29.0 ± 23.7 (28.0–69.2)	115.1 ± 167.9 (0.9–710.5)	37.2 ± 21.7 (1.5–92.9)
6 months	62.3 ± 98.8 (0.7–586.0)	51.3 ± 24.2 (6.8–92.8)	48.8 ± 69.6 (0.7–396.2)	52.5 ± 23.4 (0.3–92.8)	124.2 ± 173.8 (1.8–586.0)	45.7 ± 28.3 (6.8–80.3)
1 year	40.2 ± 64.5 (0.4–238.0)	59.1 ± 28.0 (18.6–97.2)	21.1 ± 27.0 (0.4–89.4)	65.6 ± 23.3 (0.1–97.2)	93.8 ± 103.0 (5.5–238.0)	40.9 ± 33.1 (18.6–80.1)

^a^ Mean ± standard deviation

^b^ Patient group 1, treated by USgHIFU at Center 1

^c^ Patient group 2, treated by USgHIFU at Center 2

### Funaki classification and non-perfused volume

In the presented subcohort G1, 54% of the treated fibroids were classified as Funaki type 1 (*n* = 41), 26.3% as Funaki type 2 (*n* = 20), and 19.7% as Funaki type 3 (*n* = 15). The non-perfused volume (NPV, %) was determined at the first post-intervention contrast-enhanced imaging and averaged 70.3 ± 38.2%. The mean NPV of the treated Funaki type 1 fibroids (77.4 ± 36.6%) and type 2 fibroids (77.5 ± 34.6%) were nearly identical (*p* = 0.998). However, there was a significant difference in the mean NPV of type 3 fibroids (41.6 ± 35.2%) compared to type 1 and type 2 fibroids (each *p* < 0.05). In addition, regarding all treated fibroids, 69.2% (*n* = 45) showed an ablation zone with at least 50% devascularization. Furthermore, 80% (*n* = 29) exhibited an ablation zone of at least 44.6% and 90% (*n* = 19) of at least 29.2%. Of the 19 myomas with more than 90% devascularization, *n* = 15 were Funaki type 1, and *n* = 4 were Funaki type 2.

### Clinical outcomes

**Fig. 6:**
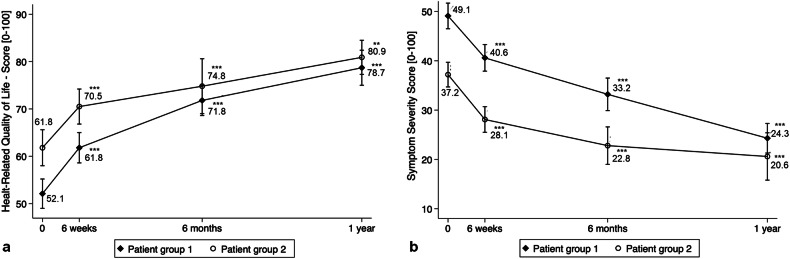
Health-related quality of life (HRQoL) (**a**) and symptom severity score (SSS) (**b**) after USgHIFU compared with baseline values in G1 and G2; *p*-values refer to comparison with baseline values (*p ≤ 0.05; **p < 0.01; ***p < 0.001)

Patients in G1 reported experiencing more severe fibroid-related symptoms (G1: 49.1 ± 19.3 vs. G2: 37.2 ± 16.1, *p* = 0.002) and lower HRQoL (G1: 52.1 ± 22.0 vs. G2: 61.8 ± 24.3, *p* = 0.045) in comparison to patients in G2. The baseline mean SSS for the entire patient cohort was 43.9 ± 18.8 (range: 3.1–84.4) and decreased significantly to 35.4 ± 18.2, 31.1 ± 20.0 and 23.1 ± 14.0 at 6 weeks, 6 months and 1 year after HIFU, respectively (each *p* < 0.001). HRQoL scores averaged 56.5 ± 23.4 before HIFU (range: 9.5–97.4) and significantly improved to 65.4 ± 22.2, 72.5 ± 19.5 and 79.4 ± 15.3 at 6 weeks, 6 months and 1 year after HIFU, respectively (each *p* < 0.001). Table 5 and Fig. 6 illustrate the post-interventional changes in SSS and HRQoL in both collectives. There was no statistically significant correlation found between the baseline SSS or HRQoL and the baseline volume of the largest uterine fiboid. In G1, USgHIFU treatment was repeated in four patients within the first year after the initial treatment session due to recurrent symptoms. During the post-interventional course, two patients underwent UAE at 24 and 40 months after USgHIFU. In addition, two other patients underwent hysterectomy, both 17 months after USgHIFU. Four patients had complication-free pregnancies and deliveries following their USgHIFU treatment.

**Table 5:** Health-related quality of life (HRQoL) and symptom severity score (SSS) at baseline and 6 weeks, 6 months and 1 year after US-guided HIFU intervention in patient group 1 and patient group 2

	Total		Patient group 1^b^		Patient group 2^c^	
	HRQoL	SSS	HRQoL	SSS	HRQoL	SSS
Baseline	56.5 ± 23.4^a^ (9.5–97.4)	43.9 ± 18.8 (3.1–84.4)	52.1 ± 22.0 (9.5–97.4)	49.1 ± 19.3 (3.1–84.4)	61.8 ± 24.3 (12.1–97.4)	37.2 ± 16.1 (9.4–71.9)
6 weeks	65.4 ± 22.2 (2.6–100)	35.4 ± 18.2 (6.3–84.4)	61.8 ± 22.0 (2.6–100)	40.6 ± 18.6 (6.3–84.4)	70.5 ± 21.7 (22.4–99.1)	28.1 ± 15.1 (6.3–62.5)
6 months	72.5 ± 19.5 (24.1–100)	31.1 ± 20.0 (0–78.1)	71.8 ± 20.0 (24.1–100)	33.2 ± 21.1 (0–78.1)	74.8 ± 18.4 (36.2–98.3)	22.8 ± 12.2 (0–37.5)
1 year	79.4 ± 15.3 (28.4–100)	23.1 ± 14.0 (6.3–56.3)	78.7 ± 17.2 (28.4–100)	24.3 ± 13.8 (6.3–56.3)	80.9 ± 11.2 (63.8–99.1)	20.6 ± 15.3 (6.3–56.3)

^a^ Mean ± standard deviation (range)

^b^ Patient group 1, treated by USgHIFU at Center 1

^c^ Patient group 2, treated by USgHIFU at Center 2

### Side effects and complications

No significant complications or lasting damage related to the risks associated with HIFU were observed during the study. Short-term, peri-interventional adverse events comprised of subcutaneous edema of the lower abdominal region (32%), temporary abdominal pain (15%), vaginal discharge (5%), and bleeding (4%). The adverse effects were self-limiting or managed symptomatically using over-the-counter medications, including non-steroidal anti-inflammatory drugs, paracetamol, and/or hyoscine butylbromide. These effects were classified as grade 1 according to the Clavien-Dindo Classification. In addition, one patient who had a significantly large fibroid experienced severe post-procedural pain following the USgHIFU procedure and received an intravenous analgesia drip for one night. Furthermore, two patients experienced symptoms associated with sciatic nerve irritation. In one patient, the symptoms resolved spontaneously, whilst in the other they persisted for up to a year and required anti-inflammatory treatment [17] (Grade II according to Clavien-Dindo Classification).

## Discussion

Ultrasound-guided high-intensity focused ultrasound (HIFU) is currently considered one of the most promising therapeutic applications of ultrasound [18]. This study includes 100 women with symptomatic uterine fibroids treated with USgHIFU. To our knowledge, it represents the first bi-centric clinical trial to report on USgHIFU outcome for symptomatic uterine fibroids at two major European HIFU centers. Both centers employed a uniform treatment approach, with physicians selecting technical parameters for each patient depending on fibroid size, type, and position in the uterus, as well as proximity to at-risk structures. Our study utilized a USgHIFU for fibroid ablation, which presents challenges in measuring applied energy in the focal zone. Unlike MRgHIFU, where temperature elevation monitors energy delivery, energy efficiency with USgHIFU is estimated. However, US-guided devices are considered more powerful than MRI-guided ones, allowing for shorter treatment durations and deeper targeting, particularly for larger myomas [19]. Unlike MRgHIFU, which requires patients to be inside an MRI scanner, positioning with US guidance is quicker and more straightforward.

With regard to fibroid volumes, there was a significant initial difference between the study groups, but no significant difference in volume reduction rate was observed, indicating comparable efficacy of USgHIFU for symptomatic uterine fibroids across both European centers. At the first post-interventional MRI (within the first week after USgHIFU), a slight volume increase was observed in 36.5% of treated fibroids, likely due to ablation-related reactive processes and tissue swelling [20].

The study shows that Funaki type 1 and 2 fibroids in G1 respond more effectively to USgHIFU treatment with regard to achieved NPV. Thus, categorizing fibroids according to the Funaki Classification is valuable for predicting procedural success, offering realistic patient expectations, and facilitating comparison of treatment outcomes. Additionally, the non-perfused volume ratio (NPV) serves as an objective predictor of treatment success, unlike subjective assessments, allowing for comparisons across patients, sessions, and trials. NPV also aids in evaluating symptom improvement and overall treatment success [21]. Hence, employing the Funaki classification and NPV determination can enhance treatment protocols and establish guidelines for patient care in HIFU treatment of symptomatic uterine fibroids.

In terms of clinical improvement, this study demonstrates that patients had significant clinical improvement post-USgHIFU treatment. Symptom severity score and health-related quality of life improved significantly as early as 6 weeks after treatment and continued to improve over the follow-up period. These findings are in line with large Asian studies (e.g., *n* = 618) [22]. It is worth noting that some patients initially had low symptom severity scores and high health-related quality of life scores, suggesting mild baseline symptoms. This may have limited the potential for significant improvement. Baseline symptom severity and health-related quality of life among uterine fibroid patients typically show moderate to severe symptoms in over two-thirds of cases [23]. Baseline data from the HIFU center in Germany (G1) is consistent with this finding, while data from the Bulgarian center (G2) showed slightly lower symptom severity despite higher fibroid volumes. Cultural differences in disease perception and experience may explain this variance [24]. Both European centers’ data highlight the emotional and psychosocial toll of uterine fibroids, with fatigue commonly reported, likely reflecting the disease’s significant burden [25]. In addition, fibroid symptoms’ severity and intensity are influenced by factors like fibroid location, shape, growth rate, and displacement of surrounding structures like the bowel, bladder and fallopian tubes. Further research is needed to accurately assess the anatomical impact of these factors and to comprehend the fibroid-related symptoms they influence. In our study, temporary adverse events post-USgHIFU include subcutaneous edema, pelvic pain, vaginal discharge, and sciatic nerve irritation, indicating its generally low-risk nature. These findings align with previously published results, confirming the low-risk nature of the procedure (e.g., 4.6%, *n* = 84/1807) [23, 26–29]. The side effects are manageable with measures like cooling and analgesics. Factors such as abdominal wall thickness, total energy, and abdominal scars influence minimal thermal damage during USgHIFU ablation, enhancing safety [30]. Patient positioning helps to minimize motion-related challenges during the procedure. To prevent complications, selecting suitable candidates, adhering to safety protocols, and continuous patient monitoring are essential. Proper patient preparation, including bowel and bladder management, optimizes the acoustic pathway and reduces risk. Anesthetics and sedatives have been extensively addressed in a prior publication [17]. Safety in USgHIFU treatment requires skilled operators to continuously monitor and make real-time adjustments to treatment parameters. Complication risks are higher for patients with relevant co-morbidities, larger fibroids, or fibroids near sensitive structures. Additionally, treatment parameters like energy level and sonication time, impact the likelihood of complications. Fortunately, complications of correctly performed HIFU procedures by experienced professionals are minimal. US-guided high-intensity focused ultrasound offers a low-risk, non-invasive therapeutic option.

This study has some limitations. The sample size of 100 patients with 119 HIFU-treated fibroids from two HIFU centers is relatively small, particularly given the varied fibroid locations and types within the uterus. Unfortunately, there was a relatively high dropout rate in the post-intervention period, resulting in incomplete data for some participants during follow-up. Additionally, certain parameters, such as NPV and Funaki type, were determined only in one group of patients (G1). Furthermore, the study lacked a control group undergoing conventional surgical treatment or uterine embolization, hindering direct comparison of clinical outcomes.

## Conclusion

In summary, our prospective, longitudinal clinical study conducted at two European centers demonstrates that non-invasive uterus-sparing USgHIFU treatment is an effective and low-risk procedure for symptom relief in patients with uterine fibroids. A significant reduction in tumor volume exceeding 50% one year post-treatment, alongside significant improvement in fibroid-associated symptoms (20.8 points) and health-related quality of life (22.9 points) during the post-interventional period. However, further multi-center, randomized trials comparing USgHIFU with conventional surgical and minimally invasive approaches for uterine fibroids are needed to provide valuable evidence and aid in the establishment of definitive guidelines.

## Abbreviations

HIFU: High-intensity focused ultrasound
HRQoL: Health-related quality of life
mL: Milliliters
MR: Magnetic resonance
NPV: Non-perfused volume
QOL: Quality of life
SSS: Symptom severity score
UAE: Uterine artery embolization
US: Ultrasound

## References

[1] Stovall DW (2001) Clinical symptomatology of uterine leiomyomas. Clin Obstet Gynecol 44:364–371 11344999 10.1097/00003081-200106000-00022

[2] Kröncke T, David M (2019) MR-guided focused ultrasound in fibroid treatment—results of the 4th Radiological-Gynecological Expert Meeting. Geburtshilfe Frauenheilkd 79:693–696 31354166 10.1055/a-0893-4752 PMC6647353

[3] Lyon PC, Rai V, Price N et al (2020) Ultrasound-guided high intensity focused ultrasound ablation for symptomatic uterine fibroids: preliminary clinical experience. Ultraschall Med 41:550–556 10.1055/a-0891-0729 31238385

[4] Zhang L, Zhang W, Orsi F et al (2015) Ultrasound-guided high intensity focused ultrasound for the treatment of gynaecological diseases: a review of safety and efficacy. Int J Hyperth 31:280–284 10.3109/02656736.2014.996790 25609456

[5] Tempany CM, Stewart EA, McDannold N et al (2003) MR imaging-guided focused ultrasound surgery of uterine leiomyomas: a feasibility study. Radiology 226:897–905 12616023 10.1148/radiol.2271020395

[6] Napoli A, Alfieri G, Andrani F et al (2021) Uterine myomas: focused ultrasound surgery. Semin Ultrasound CT MR 42:25–36 33541586 10.1053/j.sult.2020.08.001

[7] Jenne JW, Preusser T, Gunther M (2012) High-intensity focused ultrasound: principles, therapy guidance, simulations and applications. Z Med Phys 22:311–322 22884198 10.1016/j.zemedi.2012.07.001

[8] Izadifar Z, Izadifar Z, Chapman D, Babyn P (2020) An introduction to high intensity focused ultrasound: systematic review on principles, devices, and clinical applications. J Clin Med 9:460 10.3390/jcm9020460 PMC7073974 32046072

[9] Zhou YF (2011) High intensity focused ultrasound in clinical tumor ablation. World J Clin Oncol 2:8–27 21603311 10.5306/wjco.v2.i1.8 PMC3095464

[10] Marinova M, Rauch M, Schild HH, Strunk HM (2016) Novel non-invasive treatment with high-intensity focused ultrasound (HIFU). Ultraschall Med 37:46–55 26251996 10.1055/s-0035-1553318

[11] Funaki K, Fukunishi H, Funaki T et al (2007) Magnetic resonance-guided focused ultrasound surgery for uterine fibroids: relationship between the therapeutic effects and signal intensity of preexisting T2-weighted magnetic resonance images. Am J Obstet Gynecol 196:184.e181–184.e186 10.1016/j.ajog.2006.08.030 17306674

[12] Munro MG, Critchley HO, Broder MS et al (2011) FIGO classification system (PALM-COEIN) for causes of abnormal uterine bleeding in nongravid women of reproductive age. Int J Gynaecol Obstet 113:3–13 21345435 10.1016/j.ijgo.2010.11.011

[13] Coyne KS, Margolis MK, Bradley LD et al (2012) Further validation of the uterine fibroid symptom and quality-of-life questionnaire. Value Health 15:135–142 22264981 10.1016/j.jval.2011.07.007

[14] Spies JB, Coyne K, Guaou Guaou N et al (2002) The UFS-QOL, a new disease-specific symptom and health-related quality of life questionnaire for leiomyomata. Obstet Gynecol 99:290–300 11814511 10.1016/s0029-7844(01)01702-1

[15] Zhao WP, Chen JY, Zhang L et al (2013) Feasibility of ultrasound-guided high intensity focused ultrasound ablating uterine fibroids with hyperintense on T2-weighted MR imaging. Eur J Radiol 82:e43–e49 23000188 10.1016/j.ejrad.2012.08.020

[16] Clavien PA, Barkun J, de Oliveira ML et al (2009) The Clavien-Dindo classification of surgical complications: five-year experience. Ann Surg 250:187–196 19638912 10.1097/SLA.0b013e3181b13ca2

[17] Recker F, Thudium M, Strunk H et al (2021) Multidisciplinary management to optimize outcome of ultrasound-guided high-intensity focused ultrasound (HIFU) in patients with uterine fibroids. Sci Rep 11:22768 34815488 10.1038/s41598-021-02217-y PMC8611035

[18] Mindjuk I, Trumm CG, Herzog P et al (2015) MRI predictors of clinical success in MR-guided focused ultrasound (MRgFUS) treatments of uterine fibroids: results from a single centre. Eur Radiol 25:1317–1328 25510445 10.1007/s00330-014-3538-6

[19] Wang Y, Wang ZB, Xu YH (2018) Efficacy, efficiency, and safety of magnetic resonance-guided high-intensity focused ultrasound for ablation of uterine fibroids: comparison with ultrasound-guided method. Korean J Radiol 19:724–732 29962878 10.3348/kjr.2018.19.4.724 PMC6005948

[20] Wei W, Ji S (2018) Cellular senescence: molecular mechanisms and pathogenicity. J Cell Physiol 233:9121–9135 30078211 10.1002/jcp.26956

[21] Wang YJ, Zhang PH, Zhang R, An PL (2019) Predictive value of quantitative uterine fibroid perfusion parameters from contrast-enhanced ultrasound for the therapeutic effect of high-intensity focused ultrasound ablation. J Ultrasound Med 38:1511–1517 30286521 10.1002/jum.14838

[22] Lee JS, Hong GY, Park BJ, Kim TE (2015) Ultrasound-guided high-intensity focused ultrasound treatment for uterine fibroid & adenomyosis: a single center experience from the Republic of Korea. Ultrason Sonochem 27:682–687 26072367 10.1016/j.ultsonch.2015.05.033

[23] Cheung VY (2013) Sonographically guided high-intensity focused ultrasound for the management of uterine fibroids. J Ultrasound Med 32:1353–1358 23887944 10.7863/ultra.32.8.1353

[24] Murji A, Bedaiwy M, Singh SS et al (2020) Influence of ethnicity on clinical presentation and quality of life in women with uterine fibroids: results from a prospective observational registry. J Obstet Gynaecol Can 42:726–733.e721 31882290 10.1016/j.jogc.2019.10.031

[25] Ghant MS, Sengoba KS, Recht H et al (2015) Beyond the physical: a qualitative assessment of the burden of symptomatic uterine fibroids on women’s emotional and psychosocial health. J Psychosom Res 78:499–503 25725565 10.1016/j.jpsychores.2014.12.016

[26] Lee JS, Hong GY, Lee KH et al (2019) Safety and efficacy of ultrasound-guided high-intensity focused ultrasound treatment for uterine fibroids and adenomyosis. Ultrasound Med Biol 45:3214–3221 31563479 10.1016/j.ultrasmedbio.2019.08.022

[27] Peng S, Zhang L, Hu L et al (2015) Factors influencing the dosimetry for high-intensity focused ultrasound ablation of uterine fibroids: a retrospective study. Medicine (Baltimore) 94:e650 25837756 10.1097/MD.0000000000000650 PMC4554030

[28] Fan HJ, Zhang C, Lei HT et al (2019) Ultrasound-guided high-intensity focused ultrasound in the treatment of uterine fibroids. Medicine (Baltimore) 98:e14566 30855440 10.1097/MD.0000000000014566 PMC6417526

[29] Yu T, Luo J (2011) Adverse events of extracorporeal ultrasound-guided high intensity focused ultrasound therapy. PLoS One 6:e26110 22194777 10.1371/journal.pone.0026110 PMC3237413

[30] Yin N, Hu L, Xiao ZB et al (2018) Factors influencing thermal injury to skin and abdominal wall structures in HIFU ablation of uterine fibroids. Int J Hyperth 34:1298–1303 10.1080/02656736.2018.1433880 29506421

